# Supplementary material for: Effect of aging on the human myometrium at single-cell resolution
Source: Nat Commun. 2024 Jan 31;15:945. doi: 10.1038/s41467-024-45143-z (PMC10830479; doi:10.1038/s41467-024-45143-z)
Supplement: Supplementary file 1 — Supplementary Information [file 41467_2024_45143_MOESM1_ESM.pdf]

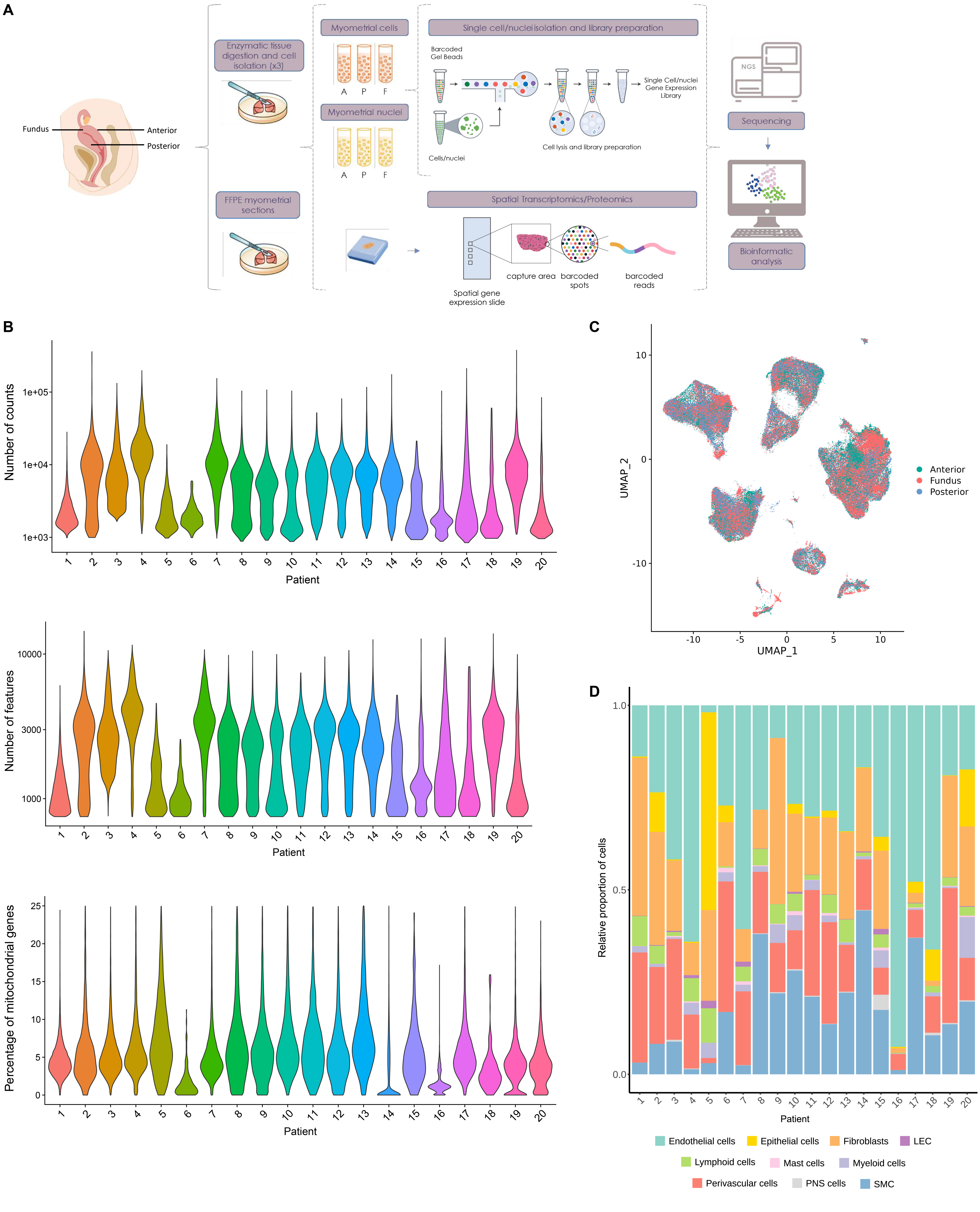

Supplementary Figure 1. Single-cell Profiling of the Human Myometrium. A) Overview of the study workflow. **B)** Violin plots illustrating the quality control metrics used in the sc/snRNA-seq analysis for each patient's features, counts, and mitochondrial percent age per cell. **C)** UMAP visualization of high-quality cells and nuclei from human myometrial samples according to the uterine region / area (anterior, posterior, fundus). **D)** Bar plots illustrate the relative contributions of myometrial cell types in each patient. Abbreviations: LEC: lymphatic endothelium; PNS: peripheral nervous system; SMC: smooth muscle cell.

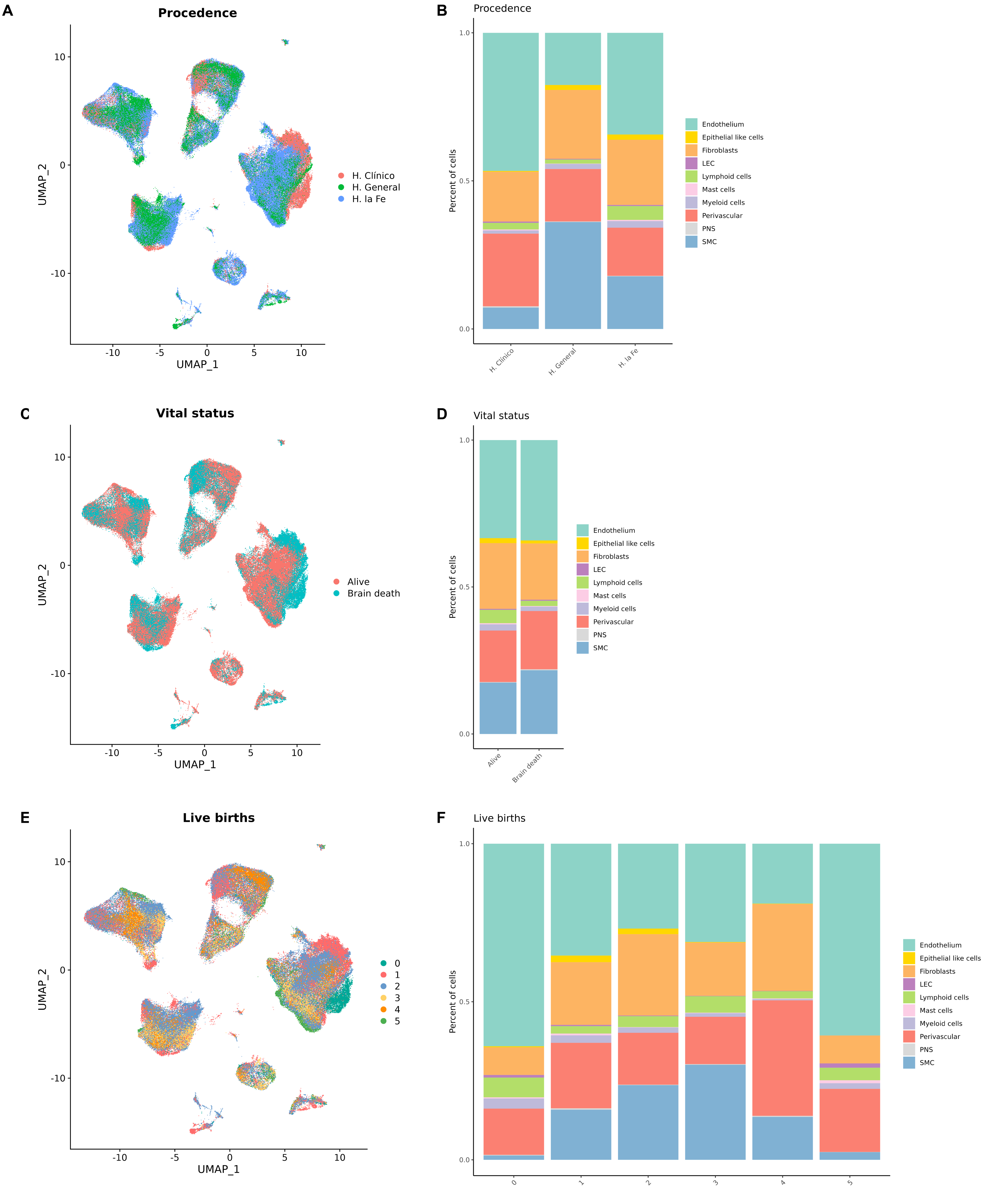

Supplementary Figure 2. UMAP visualization of high-quality cells and nuclei from human myometrial samples according to **A)** procedence or site of location, **C)** vital status, **E)** live births or parity. Bar plots illustrating ther elative contributions of myometrial cell types based on **B)** procedence or site of location, **D)** vital status and **F)** live births or parity.

A

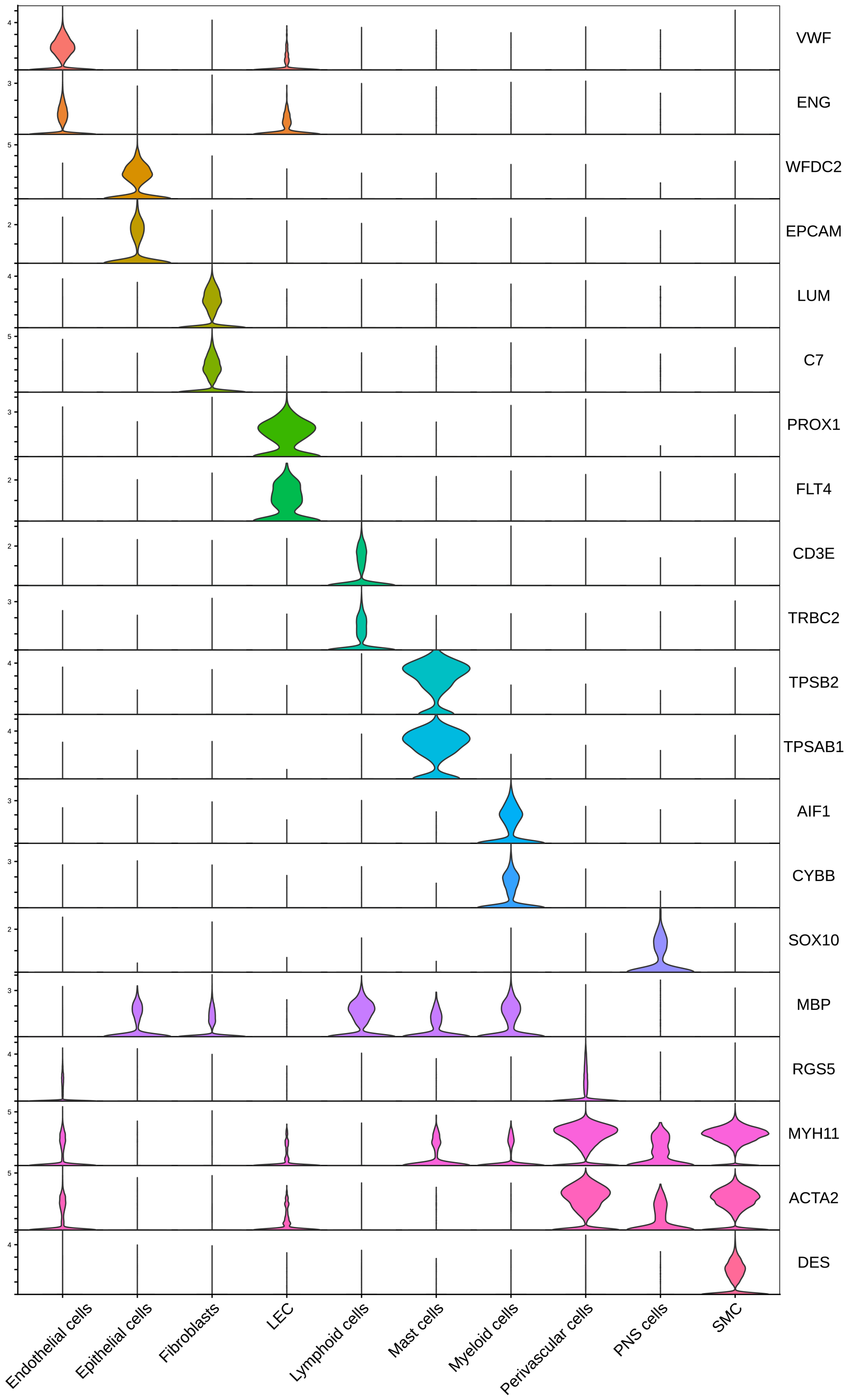

B

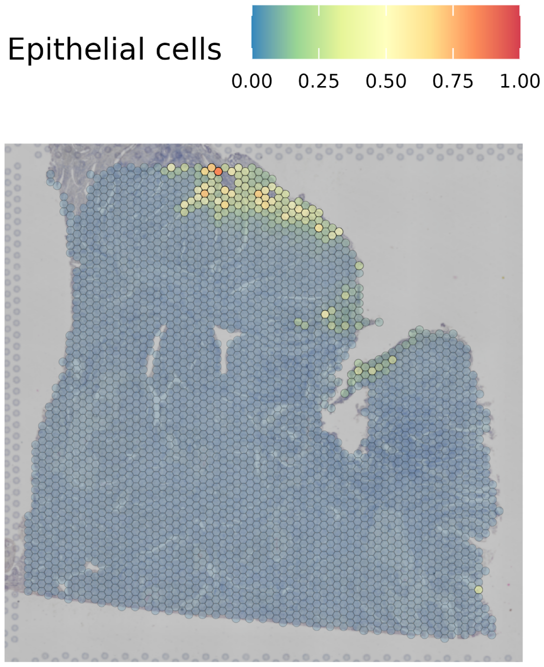

Supplementary Figure 3. Single-cell Expression of Canonical Marker Genes and the Spatial Localization of Epithelial Cells within the Myometrium. A) Violin plots reporting the expression of canonical marker genes for main cell types. **B)** Representative image of the spatial distribution of epithelial cells within the myometrial tissue (n=3 peri and 5 post-menopausal samples). Spot color indicates the proportion of epithelial cells in each spot.

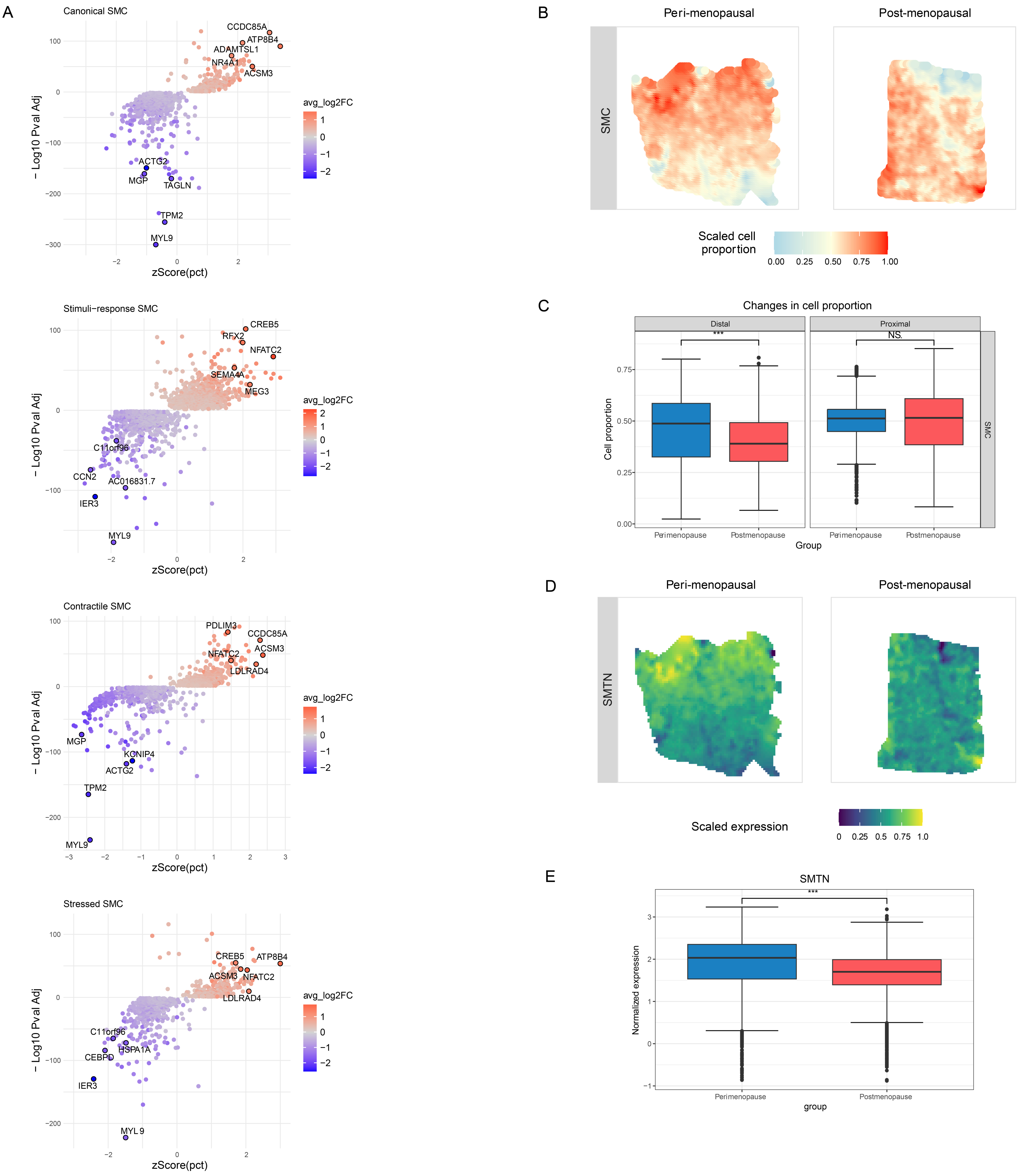

Supplementary Figure 4. Comparison of Smooth Muscle Cell Subtypes in Perimenopausal and Postmenopausal Myometria. A) Volcano plots representing differentially expressed genes in the four SMC subpopulations during myometrial aging. **B)** Representative refined spatial maps of SMCs in perimenopausal (n=3; left) and postmenopausal (n=5; right) myometrium. **C)** Boxplot of the proportion of SMCs split by group (peri vs postmenopause) and myometrial region taking the endometrium as a reference (distal or proximal). The center line shows the median for the data and error bars extend to the largest and smallest value no further than 1.5 inter-quartile range; Unpaired two-sided Wilcoxon test where ***= p value < 2.2e-16, NS=non-significant, p-value=0.7002). **D)** SMTN gene expression integrated onto a refined spatial map of the perimenopausal and postmenopausal myometrium. **E)** Boxplot of the spatial expression of the SMTN gene in the perimenopausal (n=3) and postmenopausal myometrium (n=5). The center line shows the median for the data and error bars extend to the largest and smallest value no further than 1.5 inter-quartile range; Unpaired two-sided Wilcoxon test where ***= p value < 2.2e-16. Source data are provided as a Source Data file.

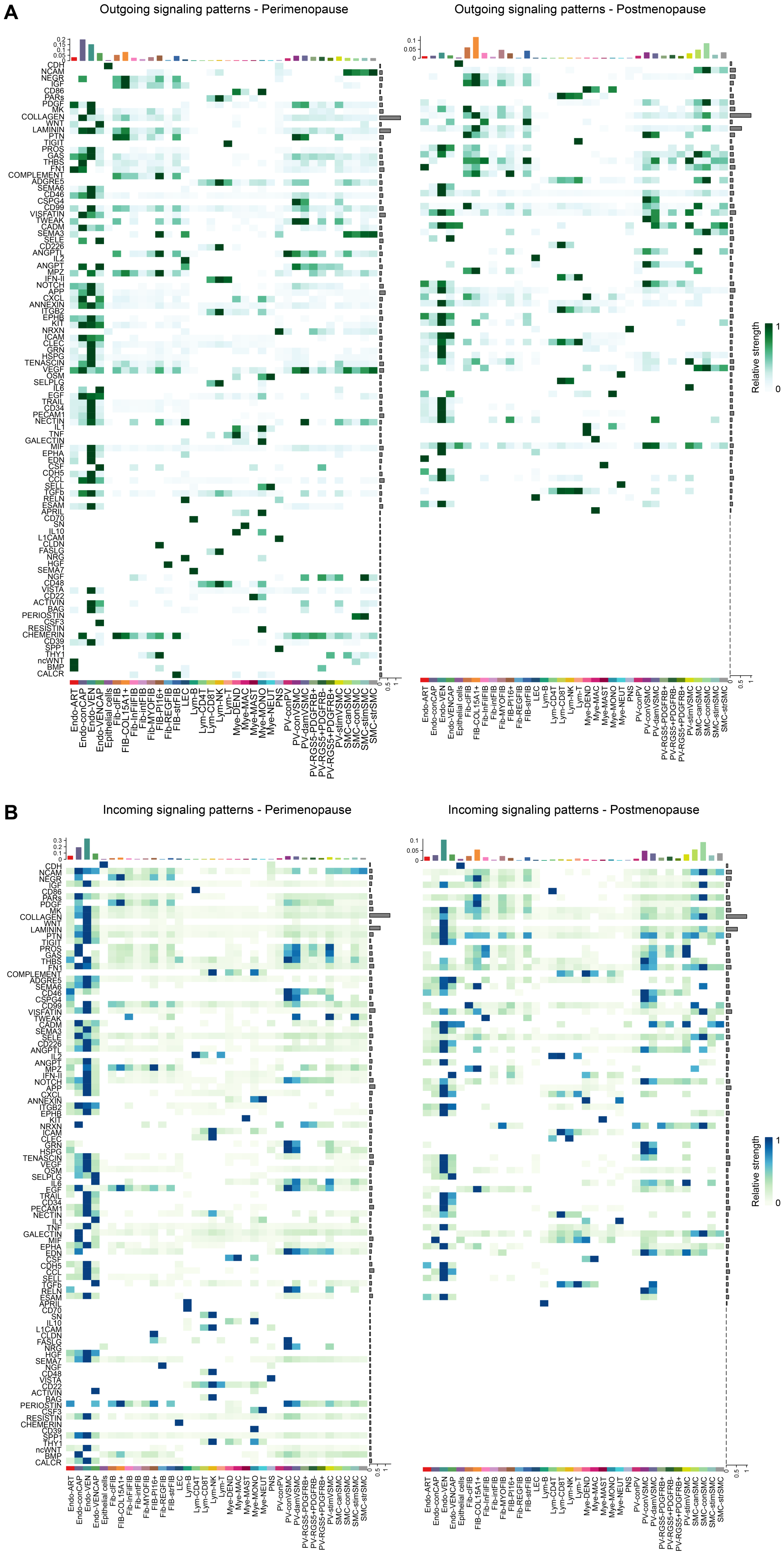

Supplementary Figure 5. Differential Age-Related Changes in Cell-to-Cell Communication in the Myometrium. A and B) Heatmaps report the contribution of signaling pathways in each cell subtype in terms of **A)** outgoing signaling and **B)** incoming signaling in the perimenopausal (left) and postmenopausal (right) myometrium. The y-axis reports the signaling pathway, while the x-axis represents the number of cells of each subtype to which the relative strength of the signal refers (top) and the subtype name displayed (bottom). The colored squares report the relative strength of each signaling pathway.

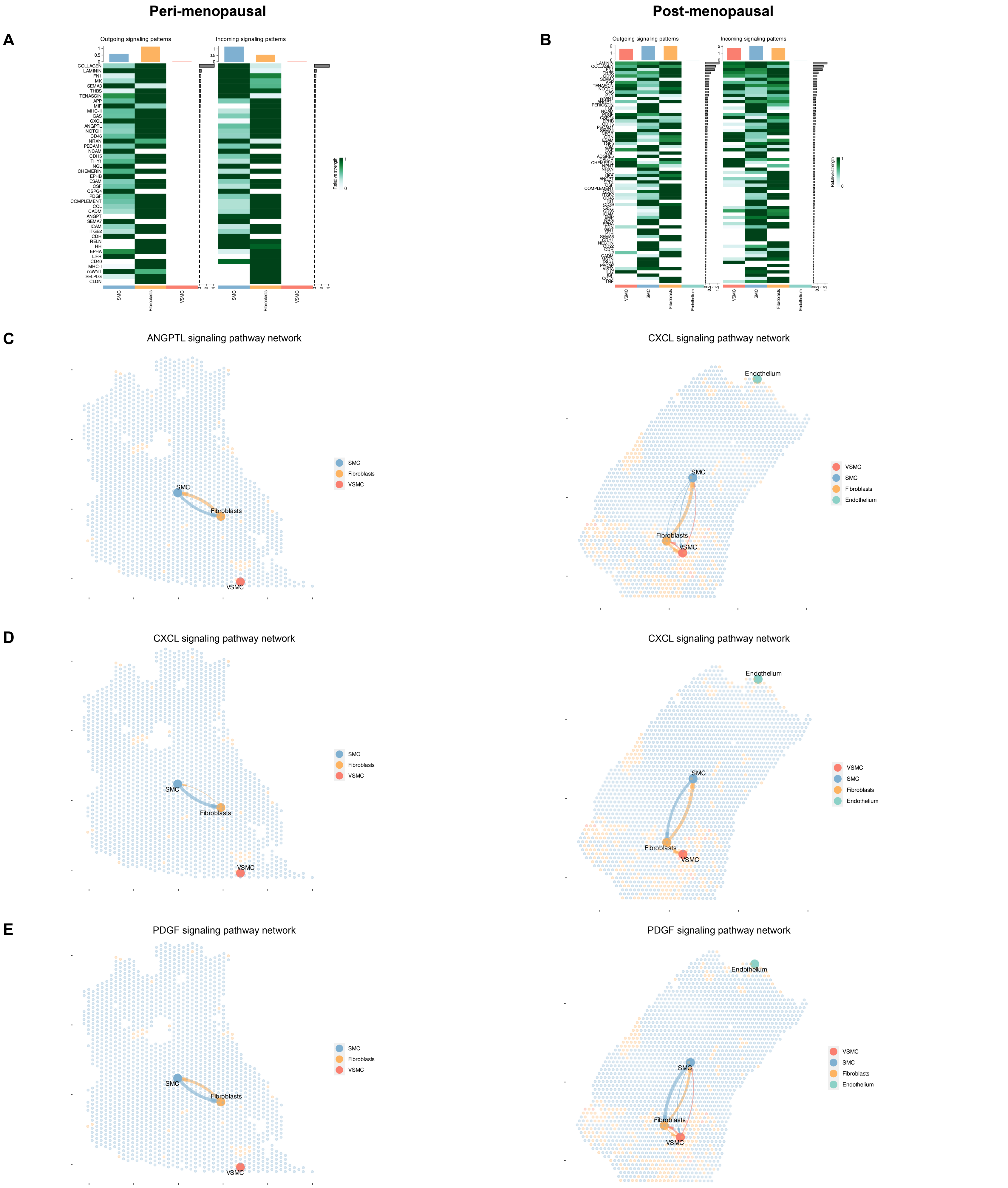

Supplementary Figure 6. Cell-to-cell communications (CCC) at spatial transcriptomic level. Heatmaps reporting the contribution of signaling pathways in each cell subtype in terms of outgoing and incoming signals in **A)** peri-menopausal (n=3) and **B)** post-menopausal representative samples (n=5). The y-axis reports the signaling pathway, while the x-axis represents the number of cells of each subtype to which the relative strength of the signal refers (top) and the subtype name displayed (bottom). The colored squares report the relative strength of each signaling pathway. **C to E)** CCC networks overlaid onto the spatial slide, where the color of spots represents the majoritarian cell population detected. Arrows between cell types are colored according to the cell type emitting the signal. The relative thickness of each line depicts the expression-based strength of the interaction between cell types in the peri-menopausal myometrium (left) and post-menopausal myometrium (right). Abbreviations: ANGPTL: angiopoietin-like; CXCL: C-X-C Motif Chemokine Ligand; PDGF: platelet-derived growth factor, SMC: Smooth muscle cell; VSMC: Vascular smooth muscle cell.

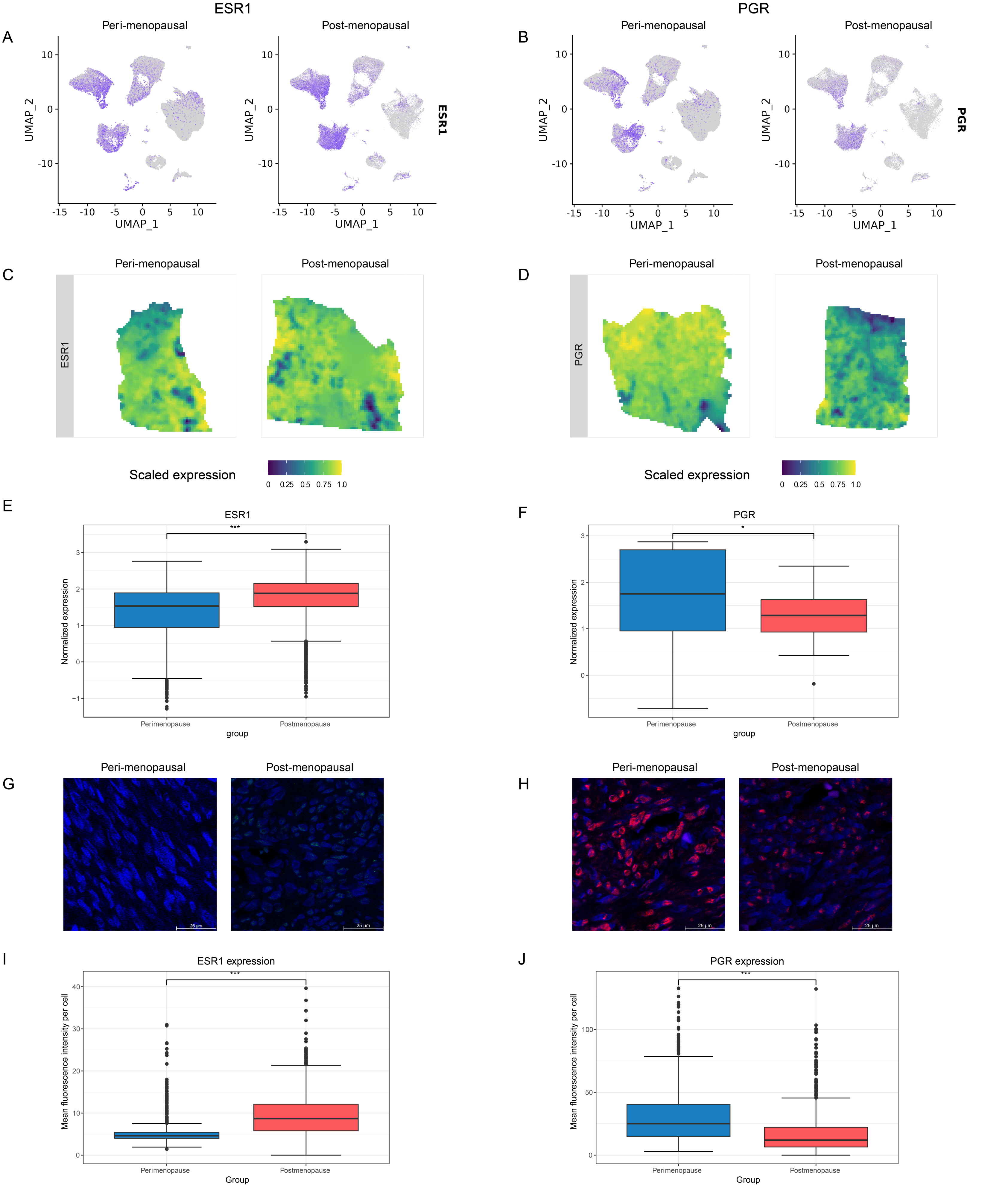

Supplementary Figure 7. Hormonal status in Perimenopausal and Postmenopausal Myometria at mRNA and protein level. Feature plots of **A)** ESR1 and **B)** PGR expression in peri and postmenopausal cells. **C)** Representative refined spatial maps of ESR1 and **D)** PGR expression in perimenopausal (n=3; eft) and postmenopausal (n=5; right) myometrium. **E)** Boxplot of ESR1 and **F)** PGR expression in peri vs postmenopause. The center line shows the median for the data and error bars extend to the largest and smallest value no further than 1.5 inter-quartile range; Unpaired two-sided Wilcoxon test where ***= p value < 2.2e-16 and *=p value=0.05508. **G)** Representative inmunofluorescence image of ESR1 (green) and **H)** PGR expression (red) in peri (n=3; left) and postmenopause (n=3; right). Scale bar= 25 µm. **I)** Quantification of immunofluorescence intensities per cell corresponding to ESR1 and **J)** PGR expression in peri and postmenopause. The center line shows the median for the data and error bars extend to the largest and smallest value no further than 1.5 inter-quartile range; Unpaired two-sided Wilcoxon test where ***= p value < 2.2e-16. Source data are provided as a Source Data file.
