# Supplementary material for: Effect of aging on the human myometrium at single-cell resolution
Source: Nat Commun. 2024 Jan 31;15:945. doi: 10.1038/s41467-024-45143-z (PMC10830479; doi:10.1038/s41467-024-45143-z)
Supplement: Supplementary file 3 — Description of Additional Supplementary Files [file 41467_2024_45143_MOESM3_ESM.pdf]

Description of Additional Supplementary Files

Supplementary Data 1. Excel file containing epidemiological, demographic, and clinicopathological outcomes of perimenopausal (n=6) and postmenopausal (n=14) women included in the study.

Supplementary Data 2. Excel file containing run summary metrics of single-cell libraries in the menopausal human myometrium.

Supplementary Data 3. Excel file containing descriptive statistics of marker genes associated with endothelial subclusters.

Supplementary Data 4. Excel file containing differentially expressed genes in endothelial subclusters during myometrial aging.

Supplementary Data 5. Excel file containing descriptive statistics of marker genes associated with fibroblast subclusters.

Supplementary Data 6. Excel file containing differentially expressed genes between fibroblasts subclusters during myometrial aging.

Supplementary Data 7. Excel file containing descriptive statistics of marker genes associated with SMC subclusters.

Supplementary Data 8. Excel file containing differentially expressed genes between smooth muscle subclusters during myometrial aging.

Supplementary Data 9. Excel file containing descriptive statistics of marker genes associated with PV subclusters.

Supplementary Data 10. Excel file containing differentially expressed genes between PV subclusters during myometrial aging.

Supplementary Data 11. Excel file containing descriptive statistics of marker genes associated with immune subclusters.

Supplementary Data 12. Excel file containing differentially expressed genes between immune subclusters during myometrial aging.

Supplementary Data 13. Excel file containing relative contributions of ligands and receptors to signaling pathways during myometrial aging.

Supplementary Data 14. Excel file containing cell counts split by patient, cell type and menopausal stage.
