# Supplementary material for: Effect of aging on the human myometrium at single-cell resolution
Source: Nat Commun. 2024 Jan 31;15:945. doi: 10.1038/s41467-024-45143-z (PMC10830479; doi:10.1038/s41467-024-45143-z)
Supplement: Supplementary file 18 — Reporting Summary [file 41467_2024_45143_MOESM18_ESM.pdf]

nature portfolio

Corresponding author(s): Aymara Mas, Carlos Simón

Last updated by author(s): Dec 12, 2023

Reporting Summary

Nature Portfolio wishes to improve the reproducibility of the work that we publish. This form provides structure for consistency and transparency in reporting. For further information on Nature Portfolio policies, see our Editorial Policies and the Editorial Policy Checklist.

Statistics

For all statistical analyses, confirm that the following items are present in the figure legend, table legend, main text, or Methods section.

n/a	Confirmed	
☐	☒	The exact sample size (*n*) for each experimental group/condition, given as a discrete number and unit of measurement
☐	☒	A statement on whether measurements were taken from distinct samples or whether the same sample was measured repeatedly
☐	☒	The statistical test(s) used AND whether they are one- or two-sided<br>*Only common tests should be described solely by name; describe more complex techniques in the Methods section.*
☐	☒	A description of all covariates tested
☐	☒	A description of any assumptions or corrections, such as tests of normality and adjustment for multiple comparisons
☐	☒	A full description of the statistical parameters including central tendency (e.g. means) or other basic estimates (e.g. regression coefficient) AND variation (e.g. standard deviation) or associated estimates of uncertainty (e.g. confidence intervals)
☐	☒	For null hypothesis testing, the test statistic (e.g. *F*, *t*, *r*) with confidence intervals, effect sizes, degrees of freedom and *P* value noted<br>*Give P values as exact values whenever suitable.*
☒	☐	For Bayesian analysis, information on the choice of priors and Markov chain Monte Carlo settings
☒	☐	For hierarchical and complex designs, identification of the appropriate level for tests and full reporting of outcomes
☒	☐	Estimates of effect sizes (e.g. Cohen's *d*, Pearson's *r*), indicating how they were calculated

Our web collection on statistics for biologists contains articles on many of the points above.

Software and code

Policy information about availability of computer code

Data collection	No software was used
Data analysis	Software, tools, algorithms and packages (including number versions) used in the study are listed hereafter:<br>CellRanger software suite (v 6.0.2); human reference genome GRCh38-2020-A; CellBender (v 0.2.0); DoubletFinder (v 2.0.3); scds tools (v 1.6.0); Seurat (v 4.1.3); clustree (R package v 0.4.4); Harmony R package (v 1.0); WebGestaltR package (v 0.4.5); miloR (v 1.6.0); Space Ranger v2.0.1; CARD package (v 1.0); CellChat R package (v 1.1.3); ImageJ (v 1.53t)

For manuscripts utilizing custom algorithms or software that are central to the research but not yet described in published literature, software must be made available to editors and reviewers. We strongly encourage code deposition in a community repository (e.g. GitHub). See the Nature Portfolio guidelines for submitting code & software for further information.

Data

Policy information about availability of data

All manuscripts must include a data availability statement. This statement should provide the following information, where applicable:

- Accession codes, unique identifiers, or web links for publicly available datasets
- A description of any restrictions on data availability
- For clinical datasets or third party data, please ensure that the statement adheres to our policy

Sequencing data supporting this study’s findings have been deposited in the Gene Expression Omnibus (GEO) under accession number GSE236660.

Research involving human participants, their data, or biological material

Policy information about studies with human participants or human data. See also policy information about sex, gender (identity/presentation), and sexual orientation and race, ethnicity and racism.

Reporting on sex and gender	Our research employs the term "women" to refer to individuals with a uterus that undergo menopause while acknowledging that not all individuals identifying as women have a uterus and/or experience menopause, and not all individuals undergoing menopause identify as women.
Reporting on race, ethnicity, or other socially relevant groupings	Not applicable
Population characteristics	This prospective, multicenter, descriptive case series included twenty females, both living and brain death donors , with ages ranging from 46 to 79 years old.
Recruitment	There was no self-selection bias in this study.<br>Samples are equal or above 46 years old due to the occurence of menopause itself, as mentioned in the introduction ("Menopause, which typically occurs between 45 and 55").<br><br>The list of samples recruited per hospital is also detailed in the methods section, as well as the clinical variables used to exclude patients from the study:<br>- The Hospital La Fe provided fifteen uteri from women undergoing hysterectomy for pelvic prolapse, while a total of five uteri (two from Hospital Clinico Universitario and three from Hospital General) were obtained from patients with brain death under the Organ Donor Program with non-cancer-related causes or traumatic injury. These five uteri were acquired through the surgical extraction of the entire uterus after meeting the criteria for brain death, following the protocol approved by the ethics committee.<br>- Patients were not considered for inclusion in the study if they had previously been diagnosed with malignancies related or unrelated to the uterus, and if such malignancies were also identified during the surgical procedure.
Ethics oversight	All procedures involving human tissue samples were approved by the Institutional Review Board of the Spanish hospitals involved: Hospital Clinico Universitario, Valencia, Spain (November 5th, 2019); Hospital La Fe, Valencia, Spain (December 4th, 2019); Hospital General Universitario, Valencia, Spain (February 12th, 2021).

Note that full information on the approval of the study protocol must also be provided in the manuscript.

Field-specific reporting

Please select the one below that is the best fit for your research. If you are not sure, read the appropriate sections before making your selection.

☒ Life sciences ☐ Behavioural & social sciences ☐ Ecological, evolutionary & environmental sciences

For a reference copy of the document with all sections, see nature.com/documents/nr-reporting-summary-flat.pdf

Life sciences study design

All studies must disclose on these points even when the disclosure is negative.

Sample size	Patients were divided into two groups: the perimenopausal group (n=6; age 46-54) and the postmenopausal (n= 15; age >54).
Data exclusions	All patients and donor families provided written informed consent, and those with gynecological disorders such as endometriosis, uterine malformations, uterine leiomyoma, endometrial polyp, hyperplasia, uterine septum, Asherman's syndrome or hydrosalpinx, were excluded from the study. Patients were not considered for inclusion in the study if they had previously been diagnosed with malignancies related or unrelated to the uterus, and if such malignancies were also identified during the surgical procedure. To mitigate the potential effects of previous infections, inclusion in the study also necessitated negative serological tests for HIV, HBV, HCV, and RPR.
Replication	We have biological replicates for all the procedures shown through the manuscript.<br>All samples within the same menopausal group (peri or postmenopause) were considered biological replicates. Thus, for the single-nuclei/single-cell experiments we had 6 biological replicates of perimenopause and 14 biological replicates of postmenopause. For spatial transcriptomics analysis we had 3 biological replicates of perimenopause and 5 biological replicates of postmenopause.<br><br>All experiments included in the study were succesful and no technical replicates were performed in this study.
Randomization	Patients were divided into two groups: the perimenopausal group (n=6; age 46-54) and the postmenopausal (n= 15; age >54). The main clinical variables (site of collection, vital status, parity or live births and c-section) that might affect the expression patterns in any cell population were also considered for further analysis.
Blinding	Blinding was not relevant in our case, since this is a descriptive study.

Reporting for specific materials, systems and methods

We require information from authors about some types of materials, experimental systems and methods used in many studies. Here, indicate whether each material, system or method listed is relevant to your study. If you are not sure if a list item applies to your research, read the appropriate section before selecting a response.

Materials & experimental systems

n/a	Involved in the study
☐	☒ Antibodies
☒	☐ Eukaryotic cell lines
☒	☐ Palaeontology and archaeology
☒	☐ Animals and other organisms
☐	☒ Clinical data
☒	☐ Dual use research of concern
☒	☐ Plants

Methods

n/a	Involved in the study
☒	☐ ChIP-seq
☒	☐ Flow cytometry
☒	☐ MRI-based neuroimaging

Antibodies

Antibodies used

Primary antibodies:
Mouse monoclonal anti-human alpha smooth muscle actin (αSMA) (Abcam, catalog# ab7817, clone# 1A4, lot# GR1009584-21; dilution 1:50)
Rabbit monoclonal anti-human voltage-dependent anion-selective channel proteins 1 and 2 (VDAC1/2) (Abcam, catalog# ab154856, clone# EPR10852(B), lot# GR219209-3 , dilution 1:50)
Mouse monoclonal anti-human estrogen receptor 1 (ESR1) (Santa Cruz Biotechnology, catalog# sc-8002, clone# F-10, lot# G0908, dilution 1:50)
Rabbit monoclonal anti-human progesterone receptor (PGR), (Abcam, catalog# ab32085, clone# YR85, lot# GR237843-14, dilution 1:50)

Secondary antibodies:
Donkey anti-mouse AlexaFluor 488 (Abcam, catalog# ab150105, lot# GR3249866-4, clone# not applicable, dilution 1:1000)
Goat anti-rabbit AlexaFluor 594 (Abcam, catalog# ab150080, lot# GR3232361-2, clone# not applicable, dilution 1:1000)

Validation

Validation statements on the manufacturers' website

Clinical data

Policy information about clinical studies
All manuscripts should comply with the ICMJEguidelines for publication of clinical research and a completedCONSORT checklist must be included with all submissions.

Clinical trial registration

This study was not registered on ClinicalTrials.gov since it is a biomedical study approved by the Institutional Review Board of the Spanish hospitals involved.

Study protocol

874867-HUTER_IGX1-HUT-CS-19-07_Human uterus cell atlas: a prospective, multicenter clinical study.

Data collection

Hospital Clinico Universitario, Valencia, Spain (November 5th, 2019); Hospital La Fe, Valencia, Spain (December 4th, 2019); Hospital General Universitario, Valencia, Spain (February 12th, 2021).

Outcomes

1) Expression patterns in any cell population from clinical variables data including site of collection, vital status, parity or live births and c-section; 2) Identification of novel cell states; 3) cell to cell communication modules across time and 4) space and cell localization with ~100µm resolution.

Plants

Seed stocks	*Report on the source of all seed stocks or other plant material used. If applicable, state the seed stock centre and catalogue number. If plant specimens were collected from the field, describe the collection location, date and sampling procedures.*
Novel plant genotypes	*Describe the methods by which all novel plant genotypes were produced. This includes those generated by transgenic approaches, gene editing, chemical/radiation-based mutagenesis and hybridization. For transgenic lines, describe the transformation method, the number of independent lines analyzed and the generation upon which experiments were performed. For gene-edited lines, describe the editor used, the endogenous sequence targeted for editing, the targeting guide RNA sequence (if applicable) and how the editor was applied.*
Authentication	*Describe any authentication procedures for each seed stock used or novel genotype generated. Describe any experiments used to assess the effect of a mutation and, where applicable, how potential secondary effects (e.g. second site T-DNA insertions, mosiacism, off-target gene editing) were examined.*
